# A phenomenological study on the experiences of patient transfer from the intensive care unit to general wards

**DOI:** 10.1371/journal.pone.0254316

**Published:** 2021-07-07

**Authors:** Eun-Young Lee, Jin-Hee Park

**Affiliations:** 1 Department of Nursing, Shinsung University, Ajou University College of Nursing, Dangjin, South Korea; 2 College of Nursing Research Institute of Nursing Science, Ajou University, Suwon, South Korea; Universita degli Studi di Udine, ITALY

## Abstract

**Objectives:**

This study aimed to derive an in-depth understanding of the transfer experience of intensive care unit (ICU) patients in South Korea through a phenomenological analysis.

**Methods:**

Participants were 15 adult patients who were admitted to a medical or surgical ICU at a university hospital for more than 48 hours before being transferred to a general ward. Data were collected three to five days after their transfer to the general ward from January to December 2017 through individual in-depth interviews and were analyzed using Colaizzi’s phenomenological data analysis method, phenomenological reduction, intersubjective reduction, and hermeneutic circle. Data analysis yielded eight themes and four theme clusters related to the unique experiences of domestic ICU patients in the process of transfer to the general ward.

**Results:**

The four main themes of the patients’ transfer experiences were “hope amid despair,” “gratitude for being alive,” “recovery from suffering,” and “seeking a return to normality.”

**Conclusion:**

Our findings expand the realistic and holistic understanding from the patient’s perspective. This study’s findings can contribute to the development of appropriate nursing interventions that can support preparation and adaptation to the transfer of ICU patients.

## Introduction

In the intensive care unit (ICU), advanced, continuous intensive care is provided based on the severity of patients’ conditions, and this care is directly related to patient survival [1]. Generally, ICU patients are transferred to wards when the acute phase of the disease has passed or the physical condition has stabilized. The transition of care from the ICU to the ward usually indicates an improvement in the clinical status of a patient; however, it is also a time when patients are particularly vulnerable. The transfer from the ICU to a general ward is one of the most stressful situations in the treatment process, which can cause health problems and even a readmission to the ICU [2–4].

When healthcare professionals decide to transfer a patient from the ICU to a general ward, some patients become anxious believing that it will adversely affect recovery owing to a lack of patient monitoring systems and the nurse-to-patient ratio in the general ward compared to the ICU [5]. In these cases, transfers can cause extreme stress in patients. In contrast, some patients feel a sense of psychological security and stability, as they are liberated from a treatment environment that is exhausting due to disturbances from noise and lighting in the ICU, family isolation, and the application of a protective guard [3]. Patients’ experience of transfer differ, and the variance can affect their health and prognosis differently [4]. Therefore, patient-centered transition care, such as providing prior guidance on planning and timing of the transfer to the general ward and evaluation of relocation stress to reduce patients’ stress and anxiety, is essential.

In the United States and Europe, various studies on the experience of transferring rooms from ICU to general wards have been conducted with patients, families, and nurses [6–8]. Based on these findings, numerous studies have been conducted to understand the development of transfer nursing interventions, the role of nurses involved in the transfer, and long-term follow-up on the quality of life after discharge [9–11]. Regarding clinical sites, the post-ICU, step-down unit, high dependency unit, and high care unit are in operation to promote the recovery and adaptation of transferred critical patients [12]. However, in South Korea, most prior studies related to the transfer of ICU patients [13, 14] focused on the concepts of transfer anxiety and stress, indicating a lack of a comprehensive understanding of patients’ experiences related to the transfer. To develop effective transfer nursing programs and interventions, empirical studies on how patients experience transfer must be conducted.

Transferring ICU patients is a common responsibility of nurses, and occurs during disease treatment and recovery [15]. In the existing literature, patients’ perception of transfer from the ICU to the general ward has not been thoroughly investigated. Therefore, this qualitative study used a phenomenological approach to elucidate the lived experiences of patients to better understand the unique transfer experiences of ICU patients and the physical and emotional experiences according to situational change [16]. Phenomenology is a research method that explores how human perceive the world [17], and can be used to provide insights into a patient’s experiences of transfer to general wards without prejudice. This study was designed to explore and describe the lived experiences of patients following transfer from the ICU to the general ward, attempting to derive uniqueness from narrative accounts of lived experience.

## Materials and methods

### Study design

This study applied Colaizzi’s phenomenological analysis method to investigate patients’ transfer experiences from the ICU to the general ward [18]. The study was performed according to the Standards for Reporting Qualitative Research [19].

### Participants

We used a purposive sampling approach [20]. Participants were 15 patients who were transferred to wards from ICUs in two university hospitals. The inclusion criteria followed the principles of Gustad et al. [21] and Strahan and Brown [22]: “patients admitted to the ICU for more than 48 hours and transferred to the ward within 3–5 days,” “adult patients over the age of 19 years,” “those who can communicate in in-depth interviews,” and “those who have not been diagnosed with neurological or psychiatric disorders that may affect their emotional response of the transfer experience or who do not take related therapeutic drugs.” Demographic and disease-related characteristics of participants are presented in Table 1.

**Table 1:** Demographic and disease-related characteristics of participants (N = 15).

No.	Gender/Age	Marital status	Employed (Yes/No)	Diagnosis	ICU			General ward		
					Nursing workload: ICU admission (WMSCN class)	Nursing workload: Transfer from ICU (WMSCN class)	Length of ICU stay (days)	Nurse to Patient ratio	Family openness	Room type
1	M/53	Married	Yes	Refractory ITP	5	4	8	1:15	Open	2
2	F/55	Married	No	AGC, ARF	5	4	4	1:15	Open	2
3	F/29	Single	No	AGC with peritoneal carcinomatosis	4	3	4	1:15	Open	1
4	F/59	Married	No	CBD stone & jejunal perforation	5	4	17	1:15	Open	4
5	F/51	Married	Yes	COPD	5	3	4	1:15	Open	4
6	M/56	Married	No	Sepsis and gastric perforation	4	3	5	1:15	Open	2
7	F/50	Married	No	Breast cancer with bone metastasis	4	2	3	1:15	Open	1
8	M/63	Married	Yes	Angina, right heart failure	4	3	3	1:15	Open	2
9	F/64	Married	Yes	Angina	4	2	3	1:15	Open	5
10	M/61	Married	No	Alcoholic liver cirrhosis, varix bleeding	4	3	3	1:15	Open	2
11	M/64	Married	No	Liver cancer with lung metastasis	5	4	8	1:15	Open	1
12	M/32	Single	Yes	STEMI	4	3	3	1:15	Open	2
13	M/48	Married	Yes	Liver cirrhosis	4	3	3	1:15	Open	2
14	M/47	Married	Yes	ARDS	5	4	6	1:15	Open	5
15	M/44	Married	Yes	Pre-exitation syndrome	3	2	3	1:15	Open	5

WMSCN, Workload Management System for Critical care Nurses; AGC, advanced gastric cancer; ARDS, acute respiratory distress syndrome; ARF, acute renal failure; CBD, common bile duct; COPD, chronic obstructive pulmonary disease; CRRT, continuous renal replacement therapy; HIPEC, hyperthermic intraperitoneal chemotherapy; ICU, intensive care unit; ITP, idiopathic thrombocytopenic purpura; STEMI, ST elevation myocardial infarction

### Data collection

The data were collected between January and December 2017 via in-depth face-to-face interviews. Before the interview, a physician at the data collection hospital introduced the participant to the researcher. Since this study was conducted among patients reporting three to five days after transfer from the ICU to a general ward, the criteria for screening participants were proposed to minimize harm to the participants and to exclude vulnerable participants. After reviewing the criteria for participant selection, the researcher explained the purpose and method of the research through a pre-interview, and confirmed the subject’s intention to participate in the study. In the main interview, the research participants’ rights protection was explained and written consent was obtained. Interviews were conducted in the general ward between three and five days after transfer to minimize distortions or omissions of participants’ experiences over time and to secure the “situational meaning” suggested by Strahan and Brown. Interview length varied from 30 to 60 minutes depending on the participant’s condition. Data collection ended when no new interpretations could be extracted and repeated statements appeared [18].

One researcher interviewed all 15 people, and each interview was audio recorded and then it was transcribed verbatim. The researcher had previous experience in qualitative research and critical care nursing, and continued to study with qualitative research societies. The researchers were not members of the hospital to avoid influencing the subject’s statements.

### Data analysis

#### Colaizzi’s descriptive phenomenological method

In-depth interview data concerning participants’ ICU transfer experiences were investigated using the phenomenological method of Colaizzi [18]. The procedure was as follows. Step 1: The recorded interview content was transcribed to prepare the study data, and the data, recorded content, and on-site records were repeatedly reviewed. Step 2: While recurrently reviewing the study data, sentences or phrases directly related to the transfer experience of ICU patients were selected and extracted as meaningful statements. Step 3: Meanings relevant to the study phenomenon were formulated by carefully considering each statement. By reiterating the process several times from Step 1 to Step 3, it was checked whether any meanings unrelated to participants’ statements were formulated or any important meanings were omitted. Step 4: From the formulated meanings, common themes were derived and organized into clusters. Step 5: Based on these results, the themes representing the study phenomenon were thoroughly and inclusively described. Step 6: Among the themes, clear and concise statements through which the key phenomenon of the study can be identified were described. Steps 2–6 were reviewed with qualitative nursing researchers and Korean literature scholars to investigate whether the derived themes and theme clusters explained the nature of participants’ initial statements, and whether the words were appropriate for explaining the research phenomenon. Step 7: Phone interviews were conducted with participants who gave their consent to participate in this process to review the validity of the study results. This procedure through the phone was performed twice in the course of confirming the study results, and it was checked across six participants to determine if the study properly captured their experiences.

#### Empirical phenomenological reduction

In this study, empirical phenomenological reduction was used in the process of creating study questions, collecting data through in-depth interviews and analysis. In particular, empirical phenomenological reduction refers to the collection of participants’ experiences regarding their natural attitudes [17]. The interview questions were as follows: (A) What did you experience while listening to the transfer plan and waiting for the actual transfer in the ICU? (B) What did you experience while transferring to the general ward from the ICU? (C) What have you experienced in the last few days after moving to the general ward from the ICU?

Additionally, bracketing was performed with six qualitative research experts throughout the study process to ensure that the researcher’s prior knowledge, experience, and natural science-oriented attitudes did not have a potential influence on study preparation, data collection and analysis, and study results that derive themes.

### Validation of results

To determine the reliability and validity of the results obtained through the study, the data were authenticated according to the evaluation criteria proposed by Guba and Lincoln [23]. First, to ensure that the researcher’s description and interpretation accurately captured reality, this study used open-ended questions during interviews so that participants’ experiences could be outlined in a natural condition, and the interview, transcription, and analysis were led and conducted in a manner to safeguard the initial statement and meaning of the participants from being compromised in the process of data collection and analysis. The researcher asked participants about their opinions and feedback on the final study results, and five professors from a nursing college and one Korean language and literature scholar were consulted regarding participants’ statements, formulated meanings, and the study results obtained from themes and theme clusters.

Subsequently, to verify whether the results could be generalized to other participants, this research adopted a qualitative sampling method that selected participants who could best inform their experiences and understanding of study problems. Data collection and analysis continued until theoretical saturation was reached, and the criteria for screening the participants and participants’ general characteristics are presented.

To ensure whether the auditability of the process of collecting data and deriving study results was conducted with consistency, this study applied the seven-step analysis process of Colaizzi [18]. Similarly, statements from the participants explaining the themes and theme clusters are provided in the study results.

Finally, to mitigate biases and maintain neutrality in the study process and results, thereby guaranteeing confirmability, the researcher attempted to comprehend participants’ experiences realistically.

### Ethical considerations

Ethical consideration and approval was obtained from the Institutional Review Board of Ajou University Hospital (AJIRB-MED-MDB-16-129). The study was conducted according to the principles expressed in the Declaration of Helsinki. Participants were provided with a full explanation and a written information sheet prior to the interview. Witten informed consent and permission to audiotape the interview sessions were obtained from all participants. The participants’ recorded and verbatim transcribed interview data were coded. The interview data and the key code list were locked away, only available to the research team.

## Results

Based on the analysis, the study participants’ transfer experiences were organized into eight theme clusters and four categories (Table 2).

**Table 2:** Transfer experience of ICU patients.

Category	Theme cluster	Theme
Hope amid despair	Message of hope through despair	Perception regarding the improvement of the illness amid uncertainty
		Hope of being alive despite extreme health status
		Hope derived from the verge of death
		Comfortable acceptance of the transfer
	Wish to escape from the ICU	Wish to be free from being called an ICU patient
		Expectation regarding the transfer decision
		Desire for a normal life
Gratitude for being alive	Gratitude for the possibility of returning to their routines	Pleasure of leaving the ICU alive
		Joy of returning to a normal life
	Appreciation for the care of medical staff and family members	Appreciation for considerate care
		A sense of security with the presence of family
		A sense of relief for continuous treatment and nursing care
Recovery from suffering	Recovery from being helpless	Regaining some independence
		Restored energy for movement
		Perceived improvement in physical symptoms
		Liberation from the uncontrollable environment
	Liberation from vulnerability	Free from having to witness the pain of other patients
		Free from the fear of witnessing the death of patients with the same condition
		Free from the feeling of isolation experienced while bedridden
Seeking a return to normality	Reflecting on their suffering	Compassion for damaged health that lived for family
		Regretting the past for negligent health behavior
		Memory about the experience of being separated from reality
		Accepting the inevitably of death
	Responsibility to return to daily life	Sense of responsibility concerning health management after hospital discharge
		Concern over the possibility of a recurring crisis
		Burden of returning to a normal life without full recovery

ICU, intensive care unit

### Category 1. Hope amid despair

#### Message of hope through despair

The formulated meanings of the theme “Message of hope through despair” were “Perception regarding the improvement of the illness amid uncertainty,” “Hope of being alive despite extremes status,” “Hope derived from the verge of death,” and “Comfortable acceptance of the transfer.” Participants comfortably accepted the transfer because they felt despair for multiple reasons, such as their body not responding to their intention, uncertainties concerning life and death, painful treatment performed in the ICU, and being in an unfamiliar environment. Therefore, participants perceived the transfer plan as a sign that their condition was improving and thus experienced the transfer as a hopeful step toward regaining control of their lives.

“There were various days where I woke up in the morning and hardly felt any improvement in my condition. I thought my dull life would end in the ICU; but when I heard the news of transfer to the ward, I felt hopeful” (Participant 6).

“When I was sick, I felt like I was going to die. But I thought I could live because I could go to the ward and had the strength to bear the pain. (Participant 6)

“When I gained consciousness and woke up in the ICU, I realized that 10 days had already passed…When I saw another critically ill patient next to me, I could not help but think about death. When I heard about moving to the ward, I had hope” (Participant 4).

"It was nice to go to the ward as I got better. I came to the hospital because my chest was tight and I couldn’t breathe, but I was feeling better when I was transferred to the ward” (Participant 5).

#### Wish to escape from the ICU

The formulated meanings of the theme “Wish to escape from the ICU” were “Wish to be free from being called an ICU patient,” “Expectation regarding the transfer decision,” and “Desire for a normal life.” participants had a negative perception concerning names such as “ICU” and “critically ill patient.” Notably, the transfer from the ICU to the general ward takes place when the health condition of the patient allows it and when the results of the main tests are satisfactory. However, the patients also mentioned that they felt frustrated and sad when the transfer was delayed because the test results did not meet the criteria. Participants longed for a transfer to return to their normal lives, such as meeting people and feeling sunlight and cool air through the window.

“When you hear the word ‘ICU’, it is a word that disheartens people. It is a place where patients without hope gather. I really hated the word ‘critical patient’. I was desperate to get out of the ICU and move to the ward as fast as possible” (Participant 2).

"I underwent a lot of blood tests to get out from the intensive care unit and to move to the ward. I was very depressed when the transfer was delayed daily because of the test results” (Participant 11).

"The time goes by slowly and there is no one in the ICU that I can talk to. I wanted to leave the intensive care unit because I wanted to talk to people as usual" (Participant 7).

### Category 2. Gratitude for being alive

#### Gratitude for the possibility of returning to their routines

The formulated meanings of the theme “Gratitude for the possibility of returning to their routines” were “Pleasure of leaving the ICU alive” and “Joy of returning to a normal life.” Some participants experienced rapid changes in health conditions, such as respiratory arrest or chest pain. Participants also expressed that they perceived this occurrence as a moving experience and did not take it for granted. Some participants had to restrain from activities or maintain a bed rest state, and they were delighted that their routines could now be partially restored. Above all, through conversations with family or other patients in the ward, they stated their experiences and concerns and listened to the experiences of other patients. This type of communication gave them a psychological sense of security.

“I underwent several near-death moments. So, on my way to this hospital, I thought to myself, ‘Will I die even before arriving at the hospital?’ or ‘Will I die during the surgery?’ and so on. One day, on the day of the transfer from the ICU, I thought ‘So I thought I would be going to heaven but now I see that I can live longer in this world’. I was ever so happy at that moment…” (Participant 9).

“I came to the ward, looked outside, and distinguished whether it was day or night… It had been a long time since I saw sunlight. Sunlight, air, wind… all these felt new.” (Participant 4)

#### Appreciation for the care of medical staff and family members

The formulated meanings of the theme “Appreciation for the care of medical staff and family members” were “Appreciation for considerate care,” “A sense of security with the presence of family,” and “A sense of relief for continuous treatment and nursing care.” Participants who spent time in the ICU and were subsequently transferred to a general ward felt grateful for the medical staff and their families who continued to provide care for them. They viewed the 24-hour care of ICU nurses in their state of total dependence as a sacrifice and, after the transfer, they felt sorry and grateful for their family members who stayed in the hospital to provide care for them. Regardless of the recovery from the disease, participants regarded their time spent in the ward with family after the transfer as valuable and meaningful. Some participants recognized the differences in the jobs of ICU nurses and general ward nurses as well as the ratio of nurses to patients; however, they perceived the difference as something natural owing to the difference in the severity of patients’ condition. They felt comfortable with the treatment and nursing care provided in the ward after the transfer and showed a sense of satisfaction and relief.

“When I was in the ICU, my lungs were filled with water, so I underwent various tests and had to use a chest tube. I didn’t have my family in the ICU, so I needed someone to rely on, like my family. Treatment would not have been possible without ICU nurses. They took care of me even when I was sleeping. Thank you very much” (Participant 1).

“Although the nurses are excellent at their jobs, it is a little hard to request for personal needs. I wanted to be with my husband. In the ward, my husband is beside me and takes care of all things without disturbing the nurses. His presence means so much to me” (Participant 7).

“The ICU nurses did a great job, but I wasn’t worried about leaving the ICU because I was conscious. The doctor in the ICU came to the ward and continued to care for me and changed my wound dressing” (Participant 4)

### Category 3. Recovery from suffering

#### Recovery from being helpless

The formulated meanings of the theme “Recovery from being helpless” were “Regaining some independence,” “Restored energy for movement,” “Perceived improvement in physical symptoms,” and “Liberation from the uncontrollable environment.” Participants acknowledged the transfer process as the first step toward becoming self-reliant and to recover from physical pain in comparison to when they could not move. After the transfer, patients actively engaged in various activities, such as managing personal hygiene on their own, recovering strength and body functions through movement, and experiencing improvement in physical symptoms.

“The day I left the ICU, I didn’t have any strength…I lied down and moved to a mobile bed with help; but I felt as if I was a courier. When I came to the ward, I was able to undergo self-rehabilitation by relaxing the muscles and joints with my wife; so, I got a lot better” (Participant 1).

“When I was in the ICU, I was kept in bed for 24 hours. …so, I never really had a chance to move on my own. After the transfer to the ward, although it is not easy and I felt pain, I tried to move little by little and do things by myself, Still, I felt that I was getting better gradually.” (Participant 6).

“I kept fasting in the ICU. I drank water for the first time after I came to the ward. So at first, I vomited, had diarrhea, and fasted again, but it gradually got better.” (Participant 13)

“In the ICU, I was conscious, but because my arms and legs were tied together, I got angry and stressed. The suffering was…crazy…Now I feel liberated from this suffering” (Participant 14)

#### Liberation from vulnerability

The formulated meanings of the theme “Liberation from vulnerability” were “Free from having to witness the pain of other patients,” “Free from the fear of witnessing the death of patients with the same condition,” and “Free from the feeling of isolation experienced while bedridden.” Participants were emotionally affected by experiencing the pain and death of others as well as their physical pain in the ICU. Seeing a dying patient made participants think about their own death, resulting in fear, dread, and sadness. Therefore, the transfer process became a psychological recovery process from negative emotional experiences. In the general ward, participants also felt psychological security and relieved their negative feelings by talking to families and other patients.

“There was a person who died when I was in the ICU and, as I was hearing the crying right next to me, it added to my fear…I was fully conscious and having to witness all those events…hearing all those noises made me scared and I could not sleep that night. I felt depressed as well…So, I was glad to be out of the ICU. I felt relieved.” (Participant 4)

“There were two people who died while I was at the ICU, and their families came and cried…how stressful it would be to see the whole process of death and finishing everything on the site. …since I also had the same disease, I could not help but feel afraid…I was feeling frightened, filled with dread, and kept thinking about death. After moving to the ward, since I no longer had to see all those painful events, I felt relieved” (Participant 8).

“Because the ICU is an isolated place and I am lying down… looking at the attending doctor is quite an atrophy in itself. Scary…”(Participant 1)

### Category 4. Seeking a return to normality

#### Reflecting on their suffering

The formulated meanings of the theme “Reflecting on their suffering” were “Compassion for damaged health that lived for family,” “Regretting the past for negligent health behavior,” “Memory about the experience of being separated from reality” and “Accepting the inevitably of death.” Participants reflected on their time in the ICU and the transfer process to the general ward. They reflected not only on the pain during the intensive treatment process, but also the discomfort they endured throughout their respective illnesses.

“There was a lot of stress at work, because it’s not easy to quit the company I’ve been working since graduating from college… I drank a lot to relieve my stress… It made my heart ache.” (Participant 15)

“I did not know about cardiac arrest or arrhythmia before. Looking back, there were several signs that warned me about my heart, but I think I ignored them. Whenever my heart beat like that, I exercised because I was tired and had alcohol and I put up with it.” (Participant 15)

“It was something like an illusion or like going around the planet; the mind went back and forth in the ICU. My body was here, but I went around in a fantasy and then I slept. . . . When I talked about it in the hospital room, people told me they experienced similar things.” (Participant 6)

“When I regained consciousness and opened my eyes, I found myself tied up…No matter how young I was, there was nothing I could do to heal my illness. The matters of being ill, life, and death felt completely beyond my control” (Participant 14).

#### Responsibility to return to daily life

The formulated meanings of the theme “Responsibility to return to daily life” were “Sense of responsibility concerning health management after hospital discharge,” “Concern over the possibility of a recurring crisis,” and “Burden of returning to a normal life without full recovery.” Participants prioritized returning to a normal life; however, they also felt burdened by habits that were difficult to control. In addition, participants actively searched and accepted information on disease and health management during this period.

“I have a family I need to take care of, so the first thing that came to mind was my family. I thought I should protect my health well because I have a family to take care of” (Participant 14).

“It’s a problem at home after being discharged. When I go home, I am worried that this crisis will happen again because I live alone” (Participant 5)

“I have to go to work as soon as I am discharged. Getting treatment in the ward was a time for relaxation. However, maybe because I vomited blood, I do not have as much strength as I had before. My job is physically demanding, hence, I have to go to work” (Participant 10).

## Discussion

Using a phenomenological approach to decipher the unique experiences of patients, in-depth interviews were conducted with 15 participants who were moved to the general ward from the ICU. Data analysis yielded eight theme clusters and four categories.

The first category of ICU transfer experience, “Hope amid despair,” highlighted that patients regard the process of planning and preparation for transfer from the ICU as hope that their condition is improving. Participants waited for the transfer, accepting the situational transition to the general ward with ease. These findings are similar to those reported by McKinney and Deeny and Odell [24, 25], who investigated the transfer experience of ICU patients. McKinney and Deeny [24] reported that patients accepted the experience of the pre-transfer stage and that leaving the ICU meant recovery and induced positive emotions, under the themes of “Acceptance” and “Desire for normality.” Further, Odell [25] reported that patients recognized the transfer as a positive step and a sign of recovery. In addition, a study by Leith [26] that examined the perception of patients and their families about the transfer, reported that 50% of patients and 60% of families viewed the transfer process as a “result of recovery” and a “positive process,” which also supports the current findings. Thus, patients perceived transfer from the ICU as positive rather than stressful, because they thought it meant that their health was improving.

The second category, “Gratitude for being alive,” shows their return to normality through the transfer. Since participants had deteriorating health, they perceived returning to their basic and normal life in a ward environment as valuable. This result is similar to the findings of a study by Strahan and Brown [22], who reported that “the hope for the independence from the dependent state,” “personal conviction to carry on with the life,” and “possibility of returning to a normal life” had an impact on the positive experience of the transfer. However, in other studies, patients have been reported to have a negative perception of the transfer experience after being transferred to a general ward, as they become aware of the lack of a monitoring system in the ward and the difference between nursing in the ward and ICU [24, 27]. These result is different from the outcomes of this study wherein the patients experienced that optimal nursing care, which was necessary for their health condition, was also provided in general wards.

These differences are related to the post-transfer family care culture in Korea. Patients were more positive about the transfer because they could be with their families. Leith et al. also reported that “the importance of family” is the main factor in the positive perceptions of the patient’s transfer [26, 28]. However, in South Korea, the burden of families participating in patients’ care after the transfer is increasing, and the caregiving culture is changing with the implementation of integrated nursing and caregiving services [29]. In foreign countries where it is difficult to expect family care, for transfer from the ICU to a general ward, a step-down unit that assists in patients’ adaptation and a transitional nursing service, such as a link nurse, are in operation [27]. Considering that changes in family care culture in South Korea, it is necessary to develop a nursing intervention program for patients who are expected to be transferred from the ICU to the general ward.

The third category, “Recovery from suffering,” highlights the physical and emotional states of the participants owing to changes in their environment. Participants recovered their reduced muscle strength and autonomy in daily life due to the transfer. After the transfer, they made efforts toward recovery by handling basic activities of daily life, such as drinking water or independently going to the toilet. In particular, most participants reported various health problems, such as pain and general weakness, which caused several negative emotions such as depression and anxiety. However, after the transfer, having conversations with family members or other meaningful people tended to bolster their psychological stability. These outcomes are similar to those from the study of McKinney and Deeny [24], which reported the transfer experience as promoting patients’ physical and mental well-being. McKinney and Deeny [24] reported “Dreams” and “Near death experiences” through the theme of “Restoring meaning” during the post-transfer process. In particular, participants in this study also repeatedly recalled the experiences of “Dreams” and “Near death experiences,” thereby giving meaning to “survival.”

Moreover, participants experienced a sense of relief by no longer witnessing emergencies and deaths in the ICU. This occurrence was a unique factor of the participants in this study; that is, it was not reported in previous studies on the transfer experience of ICU patients abroad, and it may be associated with the differences in the healthcare environment. There is a need for a psychological support program that periodically evaluates the psychological distress experienced by patients admitted to the ICU and helps them cope with the situation appropriately.

The fourth category, “Seeking a return to normality,” represents the experience of reflecting on life in relation to one’s current health status after transfer and preparing to return to daily life after discharge. In particular, unlike medical staff who regard transfer to a general ward as the next step of acute-phase treatment [1], participants prepared for the transition to be discharged and return home. Participants preparing for the discharge experienced introspection, which consisted of self-pity, reflection, and acceptance. In addition, after transferring to a general ward, patients reviewed their health-related lifestyle and habits and prepared for a crisis that could recur. Hence, there was a high demand for information and education on health behavior. As the quality of discharge education of family members participating in patient care during the transition period of critically ill patients greatly affects the discharge readiness and adaptation of the participants [30], an educational program that identifies the sense of burden experienced by patients undergoing transfer or in need of capacity building, together with the dissemination of adequate information and support, is required. Additionally, because patients who have been transferred from the ICU and discharged to their home or the local community develop a sense of social isolation while experiencing physical or mental problems, distress is a key factor affecting the quality of life of discharged patients [31, 32]. Thus, the continued development of nursing interventions and seamless reintegration is required for critical patients.

### Strengths and limitations

This study focused on patients who had been transferred from the ICU to the general ward. Although the participants were vulnerable in that they had been in the ICU within 5 days prior to the interview, they actively participated in talking about their experiences and the study reached saturation of meaning. Despite this strength, this study focused on the transfer experience of adult patients admitted to university hospitals, but other types of hospitals were not included. In addition, to capture the true experience and feelings of the patients, we did not objectively examine patients’ anxiety or relocation stress.

## Conclusion

This study employed a phenomenological approach to elucidate the transfer experiences of ICU patients in South Korea. Four core themes were found: “Hope amid despair,” “Gratitude for being alive,” “Recovery from suffering,” and “Seeking a return to normality.” The transfer experience of the participants can be described as a dynamic process of finding hope after the suffering and despair experienced in the intensive treatment process, being grateful for the fact that they survived with the help and care of various people, and preparing for the return to their daily lives and roles after the treatment-centered recovery process. Based on these results, adequate nursing interventions should be developed to support patient transfers, adaptation to life after the transfer, and discharge.

## Supporting information

S1 File Standards for Reporting Qualitative Research (SRQR). (DOCX)

S2 File Category, theme cluster, theme and sample quotes of transfer experience. (DOCX)

S3 File Interview guide. (DOCX)
