# Supplementary material for: A phenomenological study on the experiences of patient transfer from the intensive care unit to general wards
Source: PLoS One. 2021 Jul 7;16(7):e0254316. doi: 10.1371/journal.pone.0254316 (PMC8263304; doi:10.1371/journal.pone.0254316)
Supplement: S1 File — (DOCX) [file pone.0254316.s001.docx]

S1 File. Standards for Reporting Qualitative Research (SRQR)

http://www.equator-network.org/reporting-guidelines/srqr/

| No. | Topic | | Item | | check/page number |
| --- | --- | --- | --- | --- | --- |
| Title and abstract | | | | | |
| S1 | Title | | Concise description of the nature and topic of the study Identifyingthe study as qualitative or indicating the approach (e.g., ethnography,grounded theory) or data collection methods (e.g., interview, focusgroup) is recommended | | p.1 |
| S2 | abstract | | Summary of key elements of the study using the abstract format of the intended publication; typically includes background, purpose, methods, results, and conclusions | | p.2 |
| Introduction | | | | | |
| S3 | Problem formulation | | Description and significance of the problem/phenomenon studied;review of relevant theory and empirical work; problem statement | | p.2-3 |
| S4 | Purpose or research question | | Purpose of the study and specific objectives or questions | | p.3 |
| Methods | | | | | |
| S5 | Qualitative approach and research paradigm | | Qualitative approach (e.g., ethnography, grounded theory, case study,phenomenology, narrative research) and guiding theory if appropriate;identifying the research paradigm (e.g., postpositivist, constructivist/interpretivist) is also recommended; rationale | | p.3 |
| S6 | Researcher characteristics and reflexivity | | Researchers’ characteristics that may influence the research, includingpersonal attributes, qualifications/experience, relationship withparticipants, assumptions, and/or presuppositions; potential or actualinteraction between researchers’ characteristics and the researchquestions, approach, methods, results, and/or transferability | | p.5-7 |
| S7 | Context | | Setting/site and salient contextual factors; rationale | | p.4 |
| S8 | Sampling strategy | | How and why research participants, documents, or events were selected; criteria for deciding when no further sampling was necessary(e.g., sampling saturation); rationale | | p/4-5 |
| S9 | Ethical issues pertaining to human subjects | | Documentation of approval by an appropriate ethics review boardand participant consent, or explanation for lack thereof; otherconfidentiality and data security issues | | p.7 |
| S10 | Data collection methods | | Types of data collected; details of data collection procedures including(as appropriate) start and stop dates of data collection and analysis, iterative process, triangulation of sources/methods, and modification of procedures in response to evolving study findings; rationale | | p.5 |
| S11 | Data collection instruments and technologies | | Description of instruments (e.g., interview guides, questionnaires)and devices (e.g., audio recorders) used for data collection; if/how theinstrument(s) changed over the course of the study | | p.5-7 |
| **Methods** | | | | | |
| S12 | Units of study | | Number and relevant characteristics of participants, documents, or events included in the study; level of participation (could be reported in results) | | p.4-5 |
| S13 | Data processing | | Methods for processing data prior to and during analysis, includingtranscription, data entry, data management and security, verification of data integrity, data coding, and anonymization/deidentification of excerpts | | p.5-7 |
| S14 | Data analysis | | Process by which inferences, themes, etc., were identified and developed, including the researchers involved in data analysis; usually references a specific paradigm or approach; rationale | | p.6-7 |
| S15 | Techniques to enhance trustworthiness | | Techniques to enhance trustworthiness and credibility of data analysis(e.g., member checking, audit trail, triangulation); rationale | | p.6-7 |
| Results/findings | | | | | |
| S16 | | Synthesis and interpretation | Main findings (e.g., interpretations, inferences, and themes); might include development of a theory or model, or integration with prior research or theory | p.7-13 | |
| S17 | | Links to empirical data | Evidence (e.g., quotes, field notes, text excerpts, photographs) to substantiate analytic findings | p.7-13 | |
| Discussion | | | | | |
| S18 | | Integration with prior work, implications,  transferability, and contribution  (s) to the field | Short summary of main findings; explanation of how findings and conclusions connect to, support, elaborate on, or challenge conclusions of earlier scholarship; discussion of scope of application/generaliz ability; identification of unique contribution(s) to scholarship in a discipline or field | p.13--15 | |
| S19 | | Limitations | Trustworthiness and limitations of findings | p.15 | |
| Other | | | | | |
| S20 | | Conflicts of interest | Potential sources of influence or perceived influence on study conductand conclusions; how these were managed | Cover letter | |
| S21 | | Funding | Sources of funding and other support; role of funders in datacollection, interpretation, and reporting | Cover letter | |
