# Supplementary material for: A phenomenological study on the experiences of patient transfer from the intensive care unit to general wards
Source: PLoS One. 2021 Jul 7;16(7):e0254316. doi: 10.1371/journal.pone.0254316 (PMC8263304; doi:10.1371/journal.pone.0254316)
Supplement: S2 File — (DOCX) [file pone.0254316.s002.docx]

**S2 File. Category, theme cluster, theme and sample quotes of transfer experience**

| **Category** | **Theme cluster** | **Theme** | **Sample quotes** |
| --- | --- | --- | --- |
| Hope amid despair | Message of hope through despair | Perception regarding the improvement of the illness amid uncertainty  Hope of being alive despite extreme health status  Hope derived from the verge of death  Comfortable acceptance of the transfer | “When I heard the news of transfer to the ward, I felt hopeful.”  “I thought I could live because I could go to the ward.”  “When I heard about moving to the ward, I had hope.”  “I was feeling better when I was transferred to the ward” |
|  | Wish to escape from the ICU | Wish to be free from being called an ICU patient  Expectation regarding the transfer decision  Desire for a normal life | “I really hated the word ‘critical patient… I would move to the ward.”  “I was very depressed when the transfer was delayed.”  “I wanted to talk to people as usual” |
| Gratitude for being alive | Gratitude for the possibility of returning to their routines | Pleasure of leaving the ICU alive  Joy of returning to a normal life | “I was ever so happy at that moment.”  “I came to the ward, looked outside…. ..all these felt new” |
|  | Appreciation for the care of medical staff and family members | Appreciation for considerate care  A sense of security with the presence of family  A sense of relief for continuous treatment and nursing care | “Treatment would not have been possible without ICU nurses.”  “His presence means so much to me.”  “The doctor in the ICU came to the ward and continued to care for me” |
| Recovery from suffering | Recovery from being helpless | Regaining some independence  Restored energy for movement  Perceived improvement in physical symptoms  Liberation from the uncontrollable environment | “When I came to the ward, I was able to undergo self-rehabilitation.”  “After the transfer to the ward, I tried to move little by little”  “But it gradually got better.”  “I feel liberated from this suffering” |
|  | Liberation from vulnerability | Free from having to witness the pain of other patients  Free from the fear of witnessing the death of patients with the same condition  Free from the feeling of isolation experienced while bedridden | “There was a person who died when I was in the ICU .. it added to my fear.”  “Since I no longer had to see all those painful events, I felt relieved.”  “Because the ICU is an isolated place, and I am lying down” |
| Seeking a return to normality | Reflecting on their suffering | Compassion for damaged health that lived for family  Regretting the past for negligent health behavior  Memory about the experience of being separated from reality  Accepting the inevitably of death | “I drank a lot to relieve my stress... It made my heart ache.”  “There were several signs that warned me about my heart, but I ignored them.”  “It was something like an illusion or like going around the planet.”  “There was nothing I could do to heal my illness” |
|  | Responsibility to return to daily life | Sense of responsibility concerning health management after hospital discharge  Concern over the possibility of a recurring crisis  Burden of returning to a normal life without full recovery | “I should protect my health well because I have a family to take care of.”  “I am worried that this crisis will happen again.”  “My job is physically demanding, hence, I have to go to work” |
