# Supplementary material for: A phenomenological study on the experiences of patient transfer from the intensive care unit to general wards
Source: PLoS One. 2021 Jul 7;16(7):e0254316. doi: 10.1371/journal.pone.0254316 (PMC8263304; doi:10.1371/journal.pone.0254316)
Supplement: S3 File — (DOCX) [file pone.0254316.s003.docx]

| **인터뷰 가이드라인** |
| --- |

**1. Introduction (10분)**

- 인사 및 연구자 소개: 연구자 소개, 사전 면담 동의에 대한 감사인사, 연구 참여 의사 확인 후 다음 과정 진행

- 연구 목적 안내: 연구 목적과 ‘전실’ 용어 설명

- 진행방법 설명: 개별 심층 면담, 자료 분석을 위한 녹음과 기록, 예상 소요시간에 대해 설명

- 비밀보장에 대한 설명, 동의서 작성, 보상제공: 비밀보장, 자발적 참여와 철회에 대한 불이익이 없음을 안내, 동의서 작성, 금전적 보상

**2. Interview (30분 이내)**

- 연구 주제: 중환자실 환자의 전실 경험의 의미

| 질문 과정 | 적용 | 질문 |
| --- | --- | --- |
| 질문  (Opening Questions) | 긴장을 완화하고 면담 시 대상자의 역할에 대해 자신감을 갖도록 면담에 참여하게 된 이유와 간단한 전실 관련 질문으로 시작함 | Q1. 어떻게 이 면담에 참여하게 되었나요?  Q2. 언제 중환자실에서 병동으로 오셨나요?  Q3. 병동에서 간호를 도와주는 가족이나 간병인이 있나요? |
| 도입 질문  (Introductory Questions) | 참여자가 연구 주제의 방향을 알 수 있도록 주제와 관련된 쉬운 질문을 제공함 | Q4. 중환자실에서 일반병동으로 이동(전실)을 언제 알게 되었나요?  Q5. 중환자실에서 일반병동으로 이동(전실)을 누구로부터 정보를 들었나요? |
| 주 질문  (Key questions) | 개방성을 극대화하고 연구자의 경험이나 의견이 개입되지 않도록 중립적으로 설정함 | Q6. 전실 계획을 알고 중환자실에서 일반 병실로 가기 위해 기다리는 동안 무엇을 경험하셨습니까? 신체적, 정서적으로 경험한 일과 느낌을 말씀해 주십시오.  Q7. 중환자실에서 일반 병실로 이동하면서 무엇을 경험하셨습니까?  Q8. 중환자실에서 일반 병실로 이동 후 지난 며칠간 무엇을 경험하셨습니까? |
| 마무리 질문  (Ending questions) | 면담 동안 수집한 자료의 정보를 요약하여 확인하고 추가할 내용이나 마지막 표현의 기회를 제공함 | Q9. 제가 요약한 내용을 수정하거나 추가할 내용이 있으신가요?  Q10. 마지막으로 하시고 싶은 말씀이나 질문이 있으신가요? |

| **Interview guidelines** |
| --- |

**1. Introduction (10 minutes)**

- Introduction of researchers, expression of appreciation for agreeing to the pre-interview, and introduction of the next process after confirming the intention to participate.

- Explaining the purpose of the study and the term “transfer.”

- Explaining individual in-depth interviews, recording and documentation for data analysis, and estimated time required.

- Confidentiality, guidance that there was no disadvantage for voluntary participation and withdrawal, consent form and monetary compensation

**2. Interview (within 30 minutes)**

- Research Topic: Experience of patient transfer from the ICU to general ward

| Questioning process | Question |
| --- | --- |
| Opening Questions | Q1. How did you get involved in this interview?  Q2. When did you come from the intensive care unit to the ward?  Q3. Do you have a family member or caregiver to assist with nursing in the ward? |
| Introductory Questions | Q4. When did you learn about transferring from the intensive care unit to the general ward?  Q5. Who did you tell about the transfer from the intensive care unit to the general ward? |
| Key questions | Q6. Upon learning of the plan for transfer, what did you experience while waiting? Please tell us about your experiences and feelings, both physically and emotionally.  Q7. What did you experience while transferring?  Q8. What have you experienced in the past few days after transfer? |
| Ending questions | Q9. Do you have anything to edit or add to my summary?  Q10. Do you have any final inputs or questions you would like to ask? |
